# Acupuncture combined with mouse nerve growth factor in the treatment of peripheral facial palsies: systematic review and meta-analysis

**DOI:** 10.3389/fmed.2025.1657641

**Published:** 2025-08-29

**Authors:** Yufei Hu, Yijie Liu, Zhihao Diao, Yifan Jiang, Longtao Zhang, Yuxia Ma, Na Zhang

**Affiliations:** ^1^Acupuncture and Massage College, Shandong University of Traditional Chinese Medicine, Jinan, Shandong, China; ^2^Medicine Medical College of Optometry and Ophthalmology, Shandong University of Traditional Chinese Medicine, Jinan, Shandong, China; ^3^Key Laboratory of Traditional Chinese Medicine Classical Theory, Ministry of Education, Shandong University of Traditional Chinese Medicine, Jinan, Shandong, China

**Keywords:** acupuncture, meta-analysis, mouse nerve growth factor, peripheral facial palsies, systematic review

## Abstract

**Background:**

Peripheral facial palsies (PFP) had been listed as a dominant disease of acupuncture. Numerous researchers had utilized acupuncture in conjunction with mouse nerve growth factor (mNGF) for PFP. However, there is little relevant systematic and standardized evidence-based medicine, and the efficacy of this treatment modality is currently controversial.

**Objective:**

To systematically evaluate the clinical efficacy of acupuncture combined with mNGF for PFP.

**Methods:**

We systematically searched seven databases from their inception to 10 May 2024 to identify relevant randomized controlled trials (RCTs). The Cochrane Risk of Bias tool was used to evaluate the quality of the included literature, RevMan 5.4 software was used to test for heterogeneity, Stata v.15. SE software was used to perform egger test and sensitivity analysis.

**Results:**

Results from 36 RCTs involving 2,750 patients showed that compared with single therapy, combination therapy can improve scores in the overall effective rate (RR = 1.21, 95% CI [1.17, 1.25], *p* < 0.001), Facial disability index-physical (FDIP) score (MD = 3.31, 95% CI [0.01, 6.60], *p* = 0.05),facial nerve conduction velocity (MD = 0.54, 95% CI [0.02, 1.07], *p* = 0.04), Compound Muscle Action Potential (CMAP) amplitude (MD = 0.29, 95% CI [0.10, 0.48], *p* = 0.003), Portmann score (MD = 1.16, 95% CI [0.88, 1.44], *p* < 0.001) and reduce scores in Sunnybrook Facial Grading System (SFGS) static score (MD = −2.86, 95% CI [−3.89, −1.83], *p* < 0.001), House-Brackmann Facial Nerve Function Classification score (MD = −0.68, 95% CI [−1.01, −0.36], *p* < 0.001), Facial disability index-social (FDIS) score (MD = −5.26, 95% CI [−7.60, −2.91], *p* < 0.001), and R1 latency (MD = −0.58, 95% CI [−0.97, −0.18], *p* = 0.004). However, there was no significant difference in SFGS dynamic functional score.

**Conclusion:**

These findings suggest that the treatment of PFP with acupuncture combined with mNGF has a certain superiority over acupuncture alone or mNGF alone. It can be used as one of the therapeutic strategies for the treatment of PFP. However, the certainty and external authenticity of the evidence is limited due to the low quality of the methods and geographical concentration of the included studies.

**Systematic review registration:**

Identifier CRD42024548516.

## 1 Introduction

Peripheral facial palsies (PFP) is a partial or complete loss of function of some or all structures innervated by the facial nerve (i.e., cranial nerve VII), which is mainly manifested as crooked corners of the mouth on the affected side, disappearance or shallowness of the frontal lines and nasolabial folds, and paralysis of the muscles of facial expression (1). Epidemiological evidence shows that 15–23 people per 100,000 people are affected by the disease each year, with a recurrence rate of 12% (2). The precise etiology of this condition remains elusive, with widely accepted contributing factors including viral infections, tumors, trauma, and vascular pathologies (3). Some scholars believe that patients with PFP can develop pain to the face, ear, or neck through a central mechanism in which nociceptive signals are transmitted from the facial nerve to the trigeminocervical nucleus in the brainstem (4). The prevailing pathological mechanisms involve early-stage facial nerve edema, nerve compression, and impaired local circulation, accompanied by demyelination. In later stages, axonal degeneration becomes evident, with the most pronounced pathological changes localized to the stylomastoid foramen and intratemporal segments of the facial nerve (5). The co-occurrence of facial nerve palsy with trigeminal nerve involvement is potentially the most common comorbid cranial neuropathy, which will cause severe physiological discomfort to the patient with PFP (6). They are also unable to express their emotions adequately, which, together with facial aesthetic disorders, can lead to deprivation of their social functions, physiologically and psychologically impairing their quality of life (7). Therefore, it is crucial to find an effective treatment for PFP.

Acupuncture can achieve neuromodulation by inserting needles into acupuncture points, combined with various acupuncture techniques, to excite the surrounding and central nervous systems (8). PFP has been listed as a dominant disease of acupuncture (9), and studies have shown that acupuncture can promote regeneration and functional repair of damaged nerves and thus improve symptoms by up-regulating neurotrophic factors (10), enhancing the expression of adhesion molecules (11), and improving microcirculation (12), which has been widely used in the clinical treatment of PFP. Relevant systematic reviews have confirmed the evidence-based medical evidence related to the neurological mechanism of acupuncture treatment for PFP (13). Mouse nerve growth factor is a bioactive protein which can inhibit cell migration or apoptosis by binding to neuronutrient receptors and tyrosine kinase receptors, promote the growth of nerve axons and the formation of nerve plexuses by reversing the movement of neurotransmitters, and also inhibit neuronal necrosis or apoptosis caused by various nerve injuries by promoting nerve cell regeneration (14). It has the functions of protecting neurons, promoting the growth of nerve fibers and restoring nerve functions (15). Numerous researchers have utilized acupuncture in conjunction with mNGF for the treatment of PFP; nonetheless, there is a scarcity of pertinent, systematic, and standardized evidence-based medicine. At present, there is controversy over the efficacy of this treatment method (16). For this purpose, we performed a meta-analysis of randomized controlled trials (RCTs) to assess the efficacy of acupuncture combined with mNGF, drawing upon clinical evidence from various medical conditions, with a view to providing a theoretical basis for evidence-based medicine in this study.

## 2 Information and methods

This review was conducted in strict accordance with the Preferred Reporting Items for Systematic Reviews and Meta-Analyses (PRISMA) guidelines (17). This study has been registered with PROSPERO (registration number: CRD42024548516). The PRISMA checklist has been shown in Supplementary Table S1.

### 2.1 Literature search strategy

Seven databases were searched: China National Knowledge Infrastructure (CNKI), WanFang database (WanFang), VIP Database for Chinese Technical Periodicals (VIP), China Biomedical Literature Database (CBM), and Pubmed, Eabase, and Cochrane Library. The time limit for searching was from the establishment of the database to 10 May 2024. Combined with the respective characteristics of the database, the comprehensive search of subject words and free words is carried out. The English search terms include “Acupuncture” “Electroacupuncture” “Facial Nerve Palsy” “Facial Neuritis” “Facial Paralysis” “Bell’s Palsy” “Peripheral Facial Palsy” “Peripheral Facial Nerve Palsy” “Mouse Nerve Growth Factor” “Nerve Growth Factor” “Randomized” “Randomized Controlled” “RCT.” The study selection process of PubMed is taken as an example, which is shown in Table 1. At the same time, the relevant literature that may be missed will be searched manually, and the publication period of the search will be from the self-established database to May 10, 2024. A detailed search strategy can be found in Supplementary Table S2.

**Table 1:** Search strategy.

Number	Search details
#1	((((((((((((((((((((((((((peripheral facial palsies[MeSH Terms]) OR (Paralyzes, Facial)) OR (Paralysis, Facial)) OR (Facial Palsy)) OR (Facial Palsies)) OR (Palsies, Facial)) OR (Palsy, Facial)) OR (Hemifacial Paralysis)) OR (Paralyzes, Hemifacial)) OR (Facial Palsy, Lower Motor Neuron)) OR (Facial Palsy, Lower Motor Neuron)) OR (Facial Paralysis, Peripheral)) OR (Facial Paralyzes, Peripheral)) OR (Paralysis, Peripheral Facial)) OR (Peripheral Facial Paralysis)) OR (Lower Motor Neuron Facial Palsy)) OR (Facial Palsy, Upper Motor Neuron)) OR (Facial Paralysis, Central)) OR (Central Facial Paralyzes)) OR (Central Facial Paralysis)) OR (Facial Paralyzes, Central)) OR (Paralyzes, Central Facial)) OR (Paralysis, Central Facial)) OR (Upper Motor Neuron Facial Palsy)) OR (Facial Paresis)) OR (Paresis, Facial)) OR (Paresis, Facial)
#2	(((((Acupuncture[MeSH Terms]) OR (Electroacupuncture)) OR (Pharmacopuncture)) OR (acupuncture treatment)) OR (Needling)) OR (needling therapy)
#3	(Mouse nerve growth factor) OR (nerve growth factor)
#4	((((Random) OR (RCT)) OR (controlled clinical trial)) OR (randomized controlled trial)) OR (Randomized)
#5	#1 AND #2 AND #3 AND #4 AND #5

### 2.2 Inclusion criteria

(1) Study type: publicly published RCTs, regardless of whether blinding was used or whether allocation concealment was implemented. (2) Study population: confirmed patients with PFP, with no restriction on baseline characteristics such as gender, age, and disease duration but subject to comparability. (3) Interventions: acupuncture (including but not limited to acupuncture, triangular acupuncture, skin acupuncture, plum blossom acupuncture, intradermal acupuncture, fire acupuncture, awn acupuncture, electroacupuncture, warm needling, thread embedding therapy and other related acupuncture therapies, the same below) combined with mNGF (acupoint injection or intramuscular injection) in the experimental group; acupuncture or mNGF alone (acupoint injection or intramuscular injection) in the control group. (4) The outcome indexes included at least one of the following: the primary outcome index was the overall efficiency, and the secondary outcome indexes were the Sunnybrook Facial Grading System (SFGS) Score, House-Brackmann Facial Nerve Function Grading Score (H-B), Facial Disability Index Scale (FDI), facial nerve conduction velocity, Compound Muscle Action Potential (CMAP) amplitude, R1 latency, and Portmann score.

### 2.3 Exclusion criteria

(1) Non-randomized controlled trial studies such as animal experimental studies, reviews, conference proceedings, case reports, or experts’ experience summaries; (2) Not providing clear efficacy evaluation criteria; (3) Not conforming to the diagnostic criteria of PFP (4) Repeatedly published literature; (5) Literature that the full text could not be obtained; and (6) Literature with contradictory contents, confusing logic, or with obvious data errors.

### 2.4 Literature screening and data extraction

Inclusion and exclusion of all literature was carried out independently by 2 researchers, and the results were determined in consultation with a third researcher in case of disagreement. Literature management was carried out using Endnote X9 software, with cascading screening by reading titles, keywords, abstracts, and full text, and all exclusions were recorded with reasons for exclusion for further review. Data extraction was also carried out independently by two researchers and included: (1) Basic information about the included studies, including the first author, year, title, and number of participants; (2) Characteristics of the study subjects: disease duration, age, and gender; (3) Specific interventions and intervention periods; (4) Description of the specific information for each entry in the risk of bias evaluation; (5) Outcome indicators. The outcome indexes included total effective rate, the Sunnybrook Facial Grading System (SFGS) static score, House-Brackmann Facial Nerve Function Grading score, the Facial disability index (FDI) score, the facial nerve conduction velocity, the Compound Muscle Action Potential (CMAP) amplitude, R1 latency, and Portmann score.

### 2.5 Evaluation of study quality

The methodological quality of the included literature was assessed using the Risk of Bias Assessment Tool provided by the Cochrane Collaboration Network (18), and the quality profile of each RCT was independently assessed in the following seven aspects. (1) Application of randomization methods; (2) implementation of allocation concealment schemes; (3) implementation of blinding of study populations, and treatment regimen implementers; (4) implementation of blinding of outcome measures; (5) completeness of outcome data; (6) selective reporting of study results; and (7) other sources of bias (e.g., sample size estimation, baseline comparability, etc.). In the context of research, each entry’s risk is classified as “low risk” when specified conditions are fulfilled, as “high risk” when these conditions are not met, and as “uncertain” when there is insufficient information to make a definitive assessment. The final assessment of the risk of bias in the literature was conducted using established criteria, resulting in three qualitative outcomes: “high,” “low,” and “uncertain.”

### 2.6 Statistical processing

Meta-analysis was performed on the extracted data using Review Manager 5.4. Relative risk (RR) was used as the effect analysis statistic for dichotomous variables; Mean squared deviation (MD) was used as the effect analysis statistic for continuous variables, and its 95% confidence interval (CI) was calculated. If (*p* ≥ 0.05, *I*^2^ ≤ 50%), it means that the homogeneity is good, and the fixed effect model can be used for Meta-analysis, otherwise, the random effect model will be used for merging. In addition, one-way sensitivity analyses were performed to assess the stability of the study results. Visually assess publication bias by creating funnel plots through Review Manager 5.4. Stata v.15. SE was used to perform the Egger regression test for the results of three or more studies included, and a *p*-value < 0.05 was considered to be statistically significant publication bias. Finally, subgroup analyses of possible causes of heterogeneity and descriptive analyses of studies using different modeling approaches were performed.

### 2.7 GRADE assessment of the quality of the evidence

The quality of the evidence for each outcome was assessed using the GRADE (Grading of Recommendations Assessment, Development and Evaluation) evidence grading system, which was assessed in the following five dimensions: limitations, inconsistency, indirectness, imprecision, and publication bias. The quality of the evidence was classified as high, moderate, low, or very low according to the GRADE criteria (19).

## 3 Results

### 3.1 Results of literature search and screening

**Figure 1:**
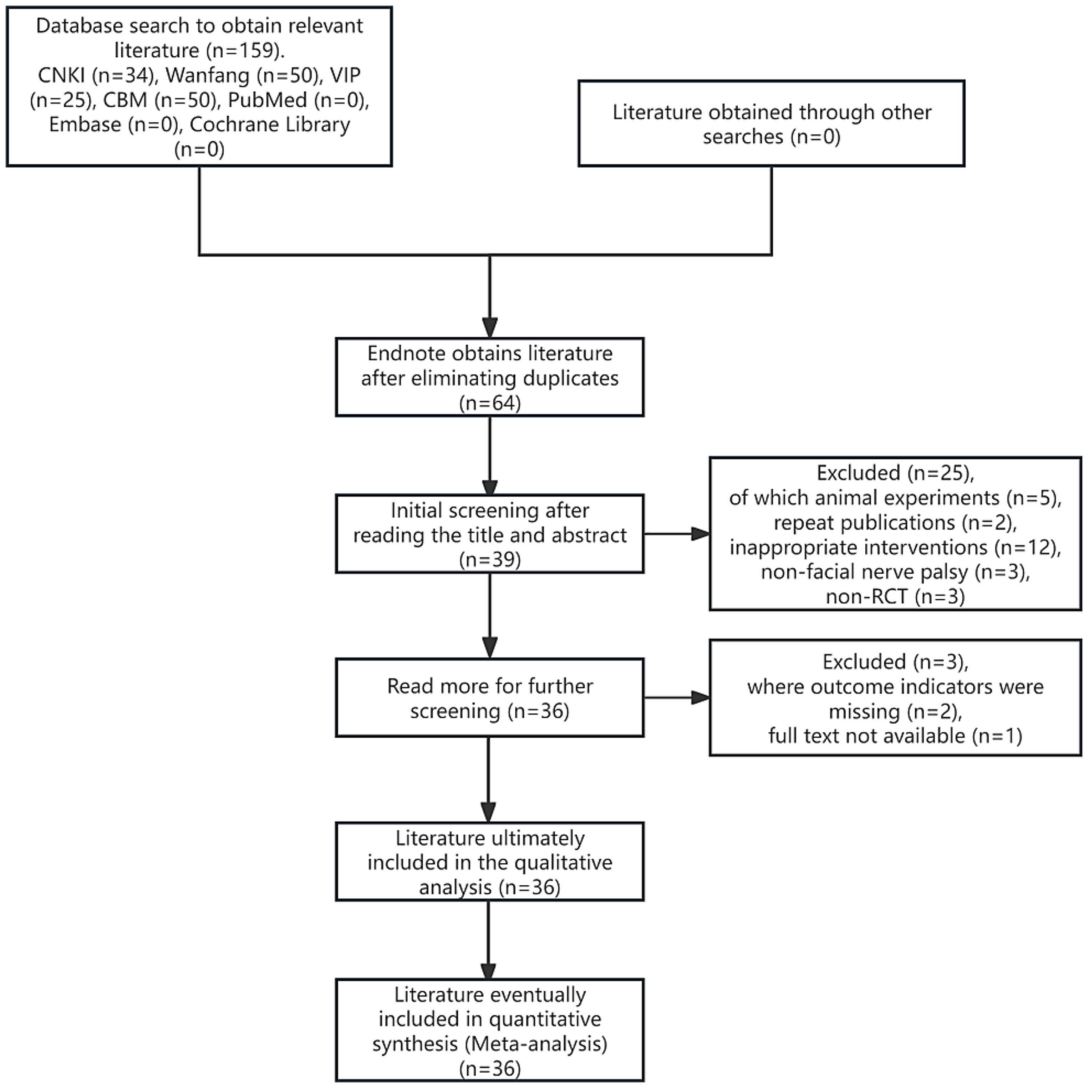
Flowchart of literature search and screening.

According to the search strategy, 159 pieces of related literature were initially retrieved, all of which were in Chinese, 64 pieces were obtained after screening out duplicates through Endnote X9 literature management software, and 36 (16, 20–54) pieces of literature were finally included after re-screening through initial screening and reading the full text, all of which were in Chinese, including 35 pieces of journal papers (16, 20–42, 45–54), and 1 piece of the dissertation article (44). The flowchart of literature search and screening is shown in Figure 1.

### 3.2 Basic information about the included studies

The 36 studies included a total of 2,750 patients, with a sample size of 1,381 cases in the experimental group and 1,369 cases in the control group. All studies were conducted in China and published in Chinese databases. The subjects met the disease diagnostic criteria for PFP. In 19 studies (13, 24–29, 31–33, 36–40, 44, 46, 47), the control group and experimental group were given basic nutritional facial nerve drug treatment; The injection methods of mNGF in 13 studies (21, 27, 30, 34, 35, 43–46, 48–50, 52) were acupoint injection and 23 (16, 20, 22–25, 28, 29, 31–33, 36–42, 47, 51, 53, 54) were intramuscular injection. Electroacupuncture was used in three studies (21, 22, 33), warm acupuncture in one study (23), and plum blossom acupuncture in one study (27). The basic features of these studies were shown in Supplementary Table S3.

### 3.3 Quality assessment of included studies

**Figure 2:**
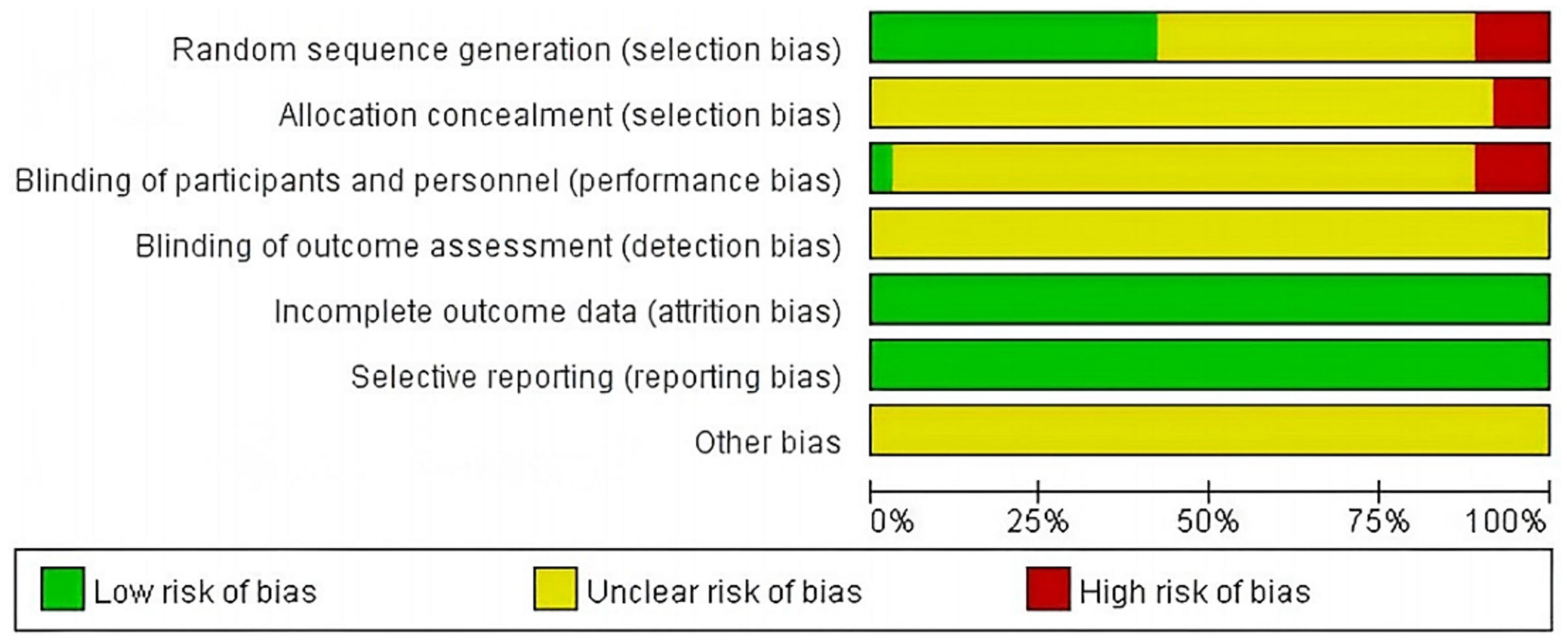
Percentage chart of risk of bias for included studies.

**Figure 3:**
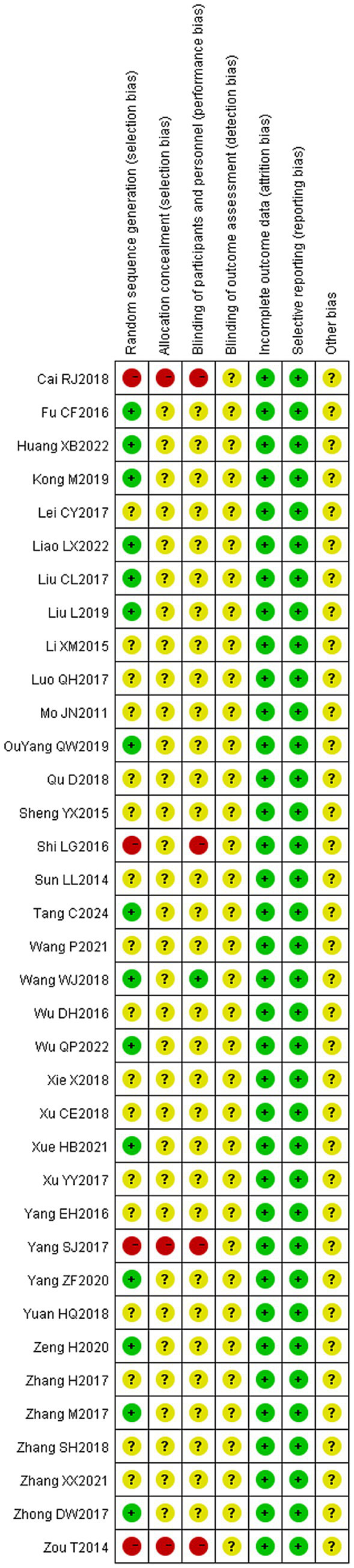
Scatterplot of risk of bias for included studies.

The 36 included literature was assessed for the risk of bias according to the bias risk assessment tool in the Cochrane Evaluation Manual. Random sequence generation: Among them, 13 (20, 21, 27, 29, 34, 38, 39, 44, 47, 50–52) used random number table method to generate random sequence, and one (42) used random drawing method to randomly group and judged as “low risk.” Three (22, 35, 49) were randomly grouped according to the order of treatment and judged as “high risk,” and four (26, 28, 29, 45) were grouped according to the treatment mode and judged as “high risk.” The remaining studies (16, 24, 25, 31–33, 36, 37, 40, 41, 43, 46, 48, 53, 54) were only described as randomized in an unknown manner and rated as “unknown risk.” Assignment hiding: Considering that grouping patients according to treatment modality (26, 28, 30, 45) would have led to the researcher predicting patient allocation, this was assessed as a “high risk,” while the rest of the literature (16, 20–25, 27, 29, 31, 44, 47–54) did not mention the use of allocation concealment, and was assessed as an “unknown risk.” Blind method: Only one (48) literature mentioned “blind method” and judged it as “low risk,” while the rest (16, 20–47, 49–54) did not mention the implementation of blind method, so it was assessed as “unknown risk.” Outcome integrity: None of the 36 literature had cases eliminated or dropped out, which was considered “low risk.” Selective reporting: The outcome indicators were fully reported in 36 literature and were judged to be “low risk.” Due to limited research data, it is not possible to determine whether there is other bias, and it is judged as “unknown risk” (see Figures 2, 3).

### 3.4 Evaluation of therapeutic efficacy

#### 3.4.1 Overall effective rate

**Figure 4:**
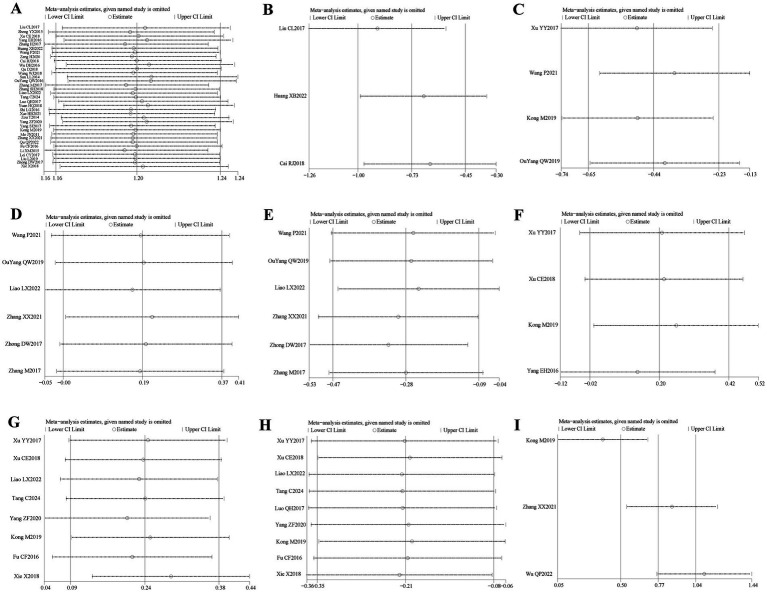
Sensitivity analysis diagram for each outcome indicator. Panel **(A)** is the total efficiency sensitivity analysis graph; **(B)** is the SFGS static score sensitivity analysis graph; **(C)** is the H-B Facial Nerve Function Grading score sensitivity analysis graph; **(D)** is the FDIP score sensitivity analysis graph; **(E)** is the FDIS score sensitivity analysis graph; **(F)** is the Facial Nerve Conduction Velocity sensitivity analysis graph; **(G)** is the CMAP amplitude sensitivity analysis graph; **(H)** is the R1 latency sensitivity analysis graph; **(I)** is the Portmann score sensitivity analysis plot.

**Figure 5:**
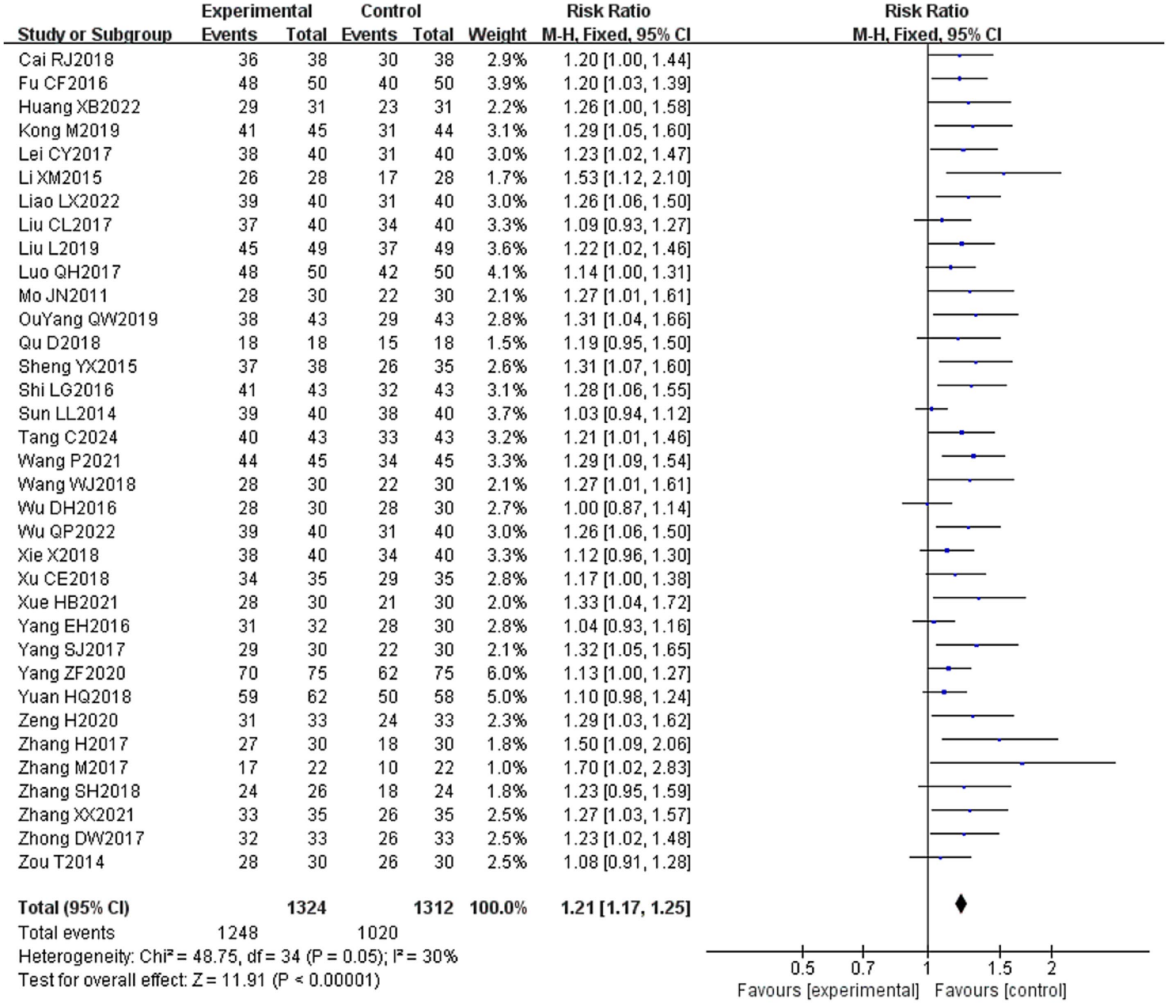
Forest plot of overall effective rate for both groups.

**Figure 6:**
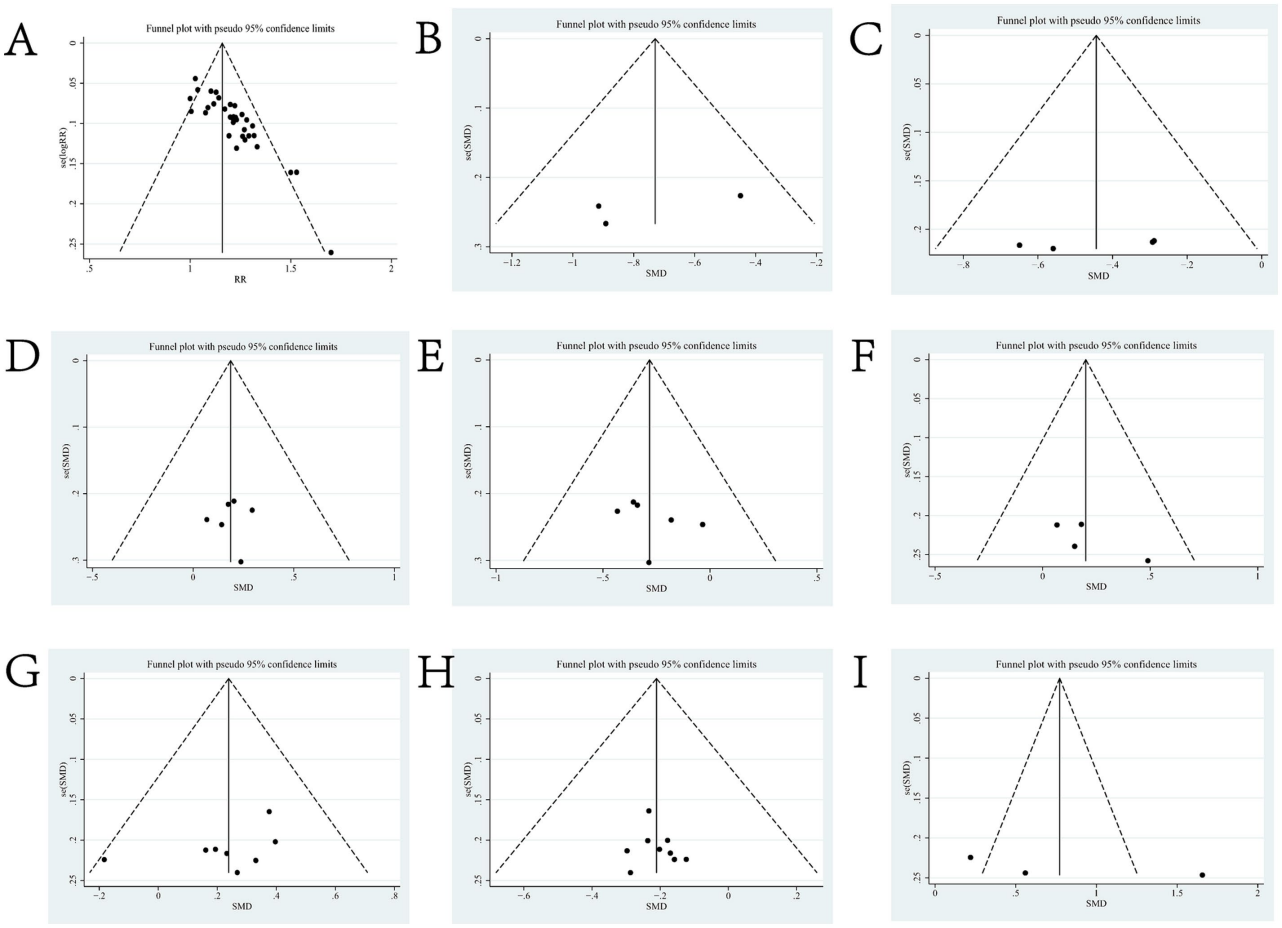
Funnel charts for outcome indicators. Panel **(A)** is the funnel plot of total efficiency; **(B)** is the funnel plot of SFGS static score; **(C)** is the funnel plot of H-B Facial Nerve Function Grading score; **(D)** is the funnel plot of FDIP score; **(E)** is the funnel plot of FDIS score; **(F)** is the funnel plot of Facial Nerve Conduction Velocity; **(G)** is the funnel plot of CMAP amplitude; **(H)** is the funnel plot of R1 Latency; and **(I)** is the Forest Plot of Portmann Score.

Thirty-five (16, 20–30, 32–54) of the 36 included literature reported overall effective rates, and a total of 2,636 patients were included. After comparing the methods of evaluating the total effective rate in each study, it was found that the criteria for determining the effective, obvious effect and cure were different in each study, but the evaluation of ineffectiveness was that there was no obvious improvement in clinical symptoms and signs after treatment, so the number of cases in the literature except for ineffectiveness was categorized as effective, which were combined and counted for meta-analysis. The heterogeneity test (*I*^2^ = 30%, *p* = 0.05) showed statistically significant findings with low heterogeneity and robust sensitivity analysis results (Figure 4A), using a fixed-effects model (Figure 5) visual assessment of the funnel plots indicated a slight publication bias (Figure 6A). The results of the meta-analysis showed that (RR = 1.21, 95% CI [1.17, 1.25], *p* < 0.001), comparing the efficacy of the experimental group with that of the control group, the difference was statistically significant, indicating that the treatment of PFP in the experimental group was clinically effective and had better efficacy than the control group. Subgroup analysis showed that: (1) grouped by age: the treatment of PFP in the experimental group was effective in children (RR = 1.17, 95% CI [1.09, 1.26], *p* < 0.001) and adults (RR = 1.21, 95% CI [1.17, 1.26], *p* < 0.001) and had better efficacy than that in the control group. (2) Grouped by injection method: Whether intramuscular injection (RR = 1.19, 95% CI [1.15, 1.24], *p* < 0.001) or acupoint injection (RR = 1.24, 95% CI [1.17, 1.32], *p* < 0.001) was used, the experimental group was effective in the treatment of PFP, and its efficacy was better than that of the control group. (3) Grouping by disease course: whether in the acute stage (RR = 1.19, 95% CI [1.25, 1.23], *p* < 0.001), recovery stage (RR = 1.15, 95% CI [1.07, 1.24], *p* = 0.0003) or sequelae stage (RR = 1.36, 95% CI [1.20, 1.55], *p* < 0.001), the experimental group was effective in treating PFP and had better efficacy than the control group. (4) Grouped according to the use of conventional drugs or not: regardless of the use of conventional drugs or not, the experimental group (with: RR = 1.19, 95% CI [1.14, 1.24], *p* < 0.001; without: RR = 1.23, 95% CI [1.17, 1.29], *p* < 0.001) was more effective than the control group in the treatment of PFP, with better efficacy than the control group. The results of subgroup analyses are shown in Table 2.

**Table 2:** Results of subgroup analyses of overall effective rate and R1 latency.

Subgroup	Overall effectiveness rate				R1 latency			
	Study	RR [95% CI]	*p-*value	*I* ^2^	Study	MD [95% CI]	*P*-value	*I* ^2^
Population	35	1.21 [1.17, 1.25]	*P* < 0.001	30%	9	−0.58 [−0.97, −0.18]	*p* < 0.01	0%
Grouped by age								
Children (≤18 years old)	6	1.17 [1.09, 1.26]	*P* < 0.001	23%	3	−0.46 [−1.16, 0.23]	*P* = 0.19	0%
Adults (18 years old)	29	1.21 [1.17, 1.26]	*P* < 0.001	28%	6	−0.55 [−1.03, −0.07]	*P* = 0.02	0%
Grouped by injection method								
Intramuscular injection	22	1.19 [1.15, 1.24]	*P* < 0.001	8%	8	−0.50 [−0.89, −0.10]	*P* = 0.01	0%
Acupoint injection	13	1.24 [1.17, 1.32]	*P* < 0.001	52%	1	−2.36 [−5.67, 0.95]	*p* = 0.16	
Grouped by disease duration								
Acute stage	23	1.19 [1.15, 1.23]	*P* < 0.001	0%	8	−0.50 [−0.89, −0.10]	*P* = 0.01	0%
Convalescence	5	1.15 [1.07, 1.24]	*P* < 0.001	71%	1	−2.36 [−5.67, 0.95]	*P* = 0.16	
Sequelae stage	4	1.36 [1.20, 1.55]	*P* < 0.001	3%				
With or without conventional drugs								
Yes	17	1.19 [1.14, 1.24]	*P* < 0.001	0%	6	−0.48 [−0.88, −0.07]	*P* = 0.02	0%
No	18	1.23 [1.17, 1.29]	*P* < 0.001	47%	3	−1.19 [−2.74, 0.36]	*p* = 0.13	0%

#### 3.4.2 Sunnybrook facial grading system (SFGS) static score

**Figure 7:**
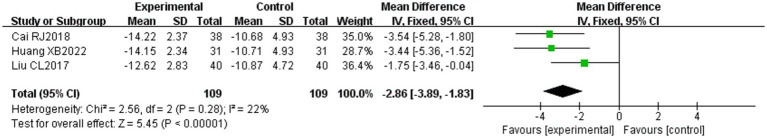
Forest plot of static SFGS scores for both groups.

Three of the 36 included literature (22, 42, 45) reported changes in SFGS static scores before and after treatment of the study participants, and a total of 218 patients were included. Heterogeneity test: (*I*^2^ = 22%, *p* = 0.28), statistically low heterogeneity among the literature, visual assessment of the funnel plots indicated a slight publication bias (Figure 6B), and Egger’s test (*p* = 0.45) demonstrated that no statistically significant publication bias was found. The results of the sensitivity analysis were robust (Figure 4B). A fixed effects model was used (Figure 7). The meta-analysis demonstrated a statistically significant difference in the reduction of SFGS static scores between the experimental group and control group (MD = −2.86, 95% CI [−3.89, −1.83], *p* < 0.001), indicating superior efficacy of the intervention of the experimental group in lowering the SFGS static scores of patients with PFP compared to the control group.

#### 3.4.3 Sunnybrook facial grading system (SFGS) dynamic score

**Figure 8:**
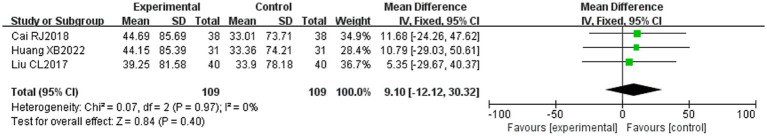
Forest plot of SFGS dynamic scores for both groups.

Three of the 36 included literature (22, 42, 45) reported changes in SFGS dynamic scores before and after treatment of study participants, and a total of 218 patients were included. Heterogeneity test: (*I*^2^ = 0%, *p* = 0.97), there was no statistical heterogeneity in the literature, and a fixed effect model was used (Figure 8). The meta-analysis demonstrated no statistically significant difference in SFGS dynamic scores changes between the two groups (MD = 9.10, 95% CI [−12.12, 30.22], *p* = 0.40), indicating comparable efficacy in improving SFGS dynamic scores between the two treatment regimens.

#### 3.4.4 House-Brackmann (H-B) facial nerve function grading score

**Figure 9:**
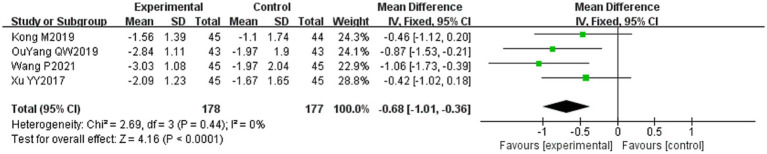
Forest plot of H-B facial nerve function grading score for both groups.

Four of the 36 included literature (31, 34, 43, 50) reported changes in the H-B facial nerve function grading before and after treatment of the study subjects, and a total of 355 patients were included. Only Ouyang QW2019 (50) scored the H-B facial nerve function grading, grade I is one point, grade II is two points, and so on, with a minimum of one point and a maximum of six points, and the higher the score indicates that the patient’s facial nerve function restoration effect is poorer, and the other three literature (31, 34, 43) described it in the way of hierarchical information, and scored the situation of the H-B facial nerve function grading in the other three literature by the same method and combined them for meta-analysis. Heterogeneity test: (*I*^2^ = 0%, *p* = 0.44), there was no statistically significant heterogeneity between the literature, visual assessment of the funnel plots demonstrated no significant publication bias (Figure 6C), and Egger’s test (*p* = 0.19) proved that no statistically significant publication bias was found. The results of the sensitivity analysis were robust (Figure 4C), using a fixed-effects model (Figure 9). Meta-analysis results showed (MD = −0.68, 95% CI [−1.01, −0.36], *p* < 0.001) a statistically significant difference in the difference in H-B facial nerve function grading scores between the experimental group and the control group, suggesting that the treatment of the experimental group was effective in reducing the H-B facial nerve function grading scores of the patients with PFP better than the control group.

#### 3.4.5 Facial disability index-physical (FDIP) scores

**Figure 10:**
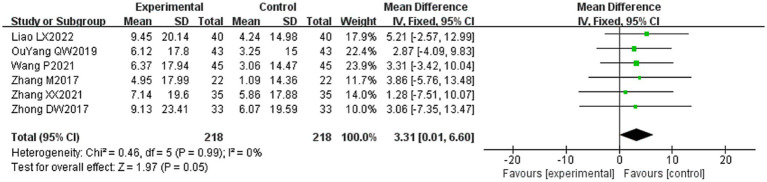
Forest plot of FDIP scores for both groups.

Six of the 36 included literature (20, 37, 43, 50–52) reported changes in FDIP scores before and after treatment of study participants, with a total of 436 patients included. Heterogeneity test: (*I*^2^ = 0%, *p* = 0.99), there was no statistically significant heterogeneity among the literature, visual assessment of the funnel plots indicated a slight publication bias (Figure 6D), and Egger’s test (*p* = 0.96) demonstrated that no statistically significant publication bias was found. The results of the sensitivity analyses were robust (Figure 4D). Using a fixed-effects model (Figure 10), the meta-analysis results demonstrated a statistically significant difference in the FDIP score changes between the experimental and control groups (MD = 3.31, 95% CI [0.01, 6.60], *p* = 0.05), indicating that the experimental group exhibited superior efficacy in improving FDIP scores among patients with PFP compared to the control group. Subgroup analysis showed that there was no statistical significance in any of the groups (Table 3).

**Table 3:** Results of subgroup analyses of FDIP score and FDIS score.

Subgroup	FDIP score				FDIS score			
	Study	MD [95% CI]	*P-*value	*I* ^2^	Study	MD [95% CI]	*P-*value	*I* ^2^
Population	6	3.31 [0.01, 6.60]	*P* = 0.05	0%	6	−2.78 [−4.46, −1.10]	*P* < 0.001	64%
Grouped by age								
Children (≤18 years old)	0				0			
Adults (18 years old)	6	3.31 [0.01, 6.60]	*P* = 0.05	0%	6	−2.78 [−4.46, −1.10]	*P* < 0.001	64%
Grouped by injection method								
Intramuscular injection	7	3.38 [−1.70, 8.47]	*P* = 0.19	0%	3	−1.10 [−3.13, 0.93]	*p* = 0.29	38%
Acupoint injection	1	3.25 [−1.07, 7.57]	*P* = 0.14	0%	3	−3.81 [−6.80, −0.82]	*P* = 0.01	0%
Grouped by disease duration								
Acute stage	2	3.48 [−2.34, 9.31]	*P* = 0.24	0%	2	−3.40 [−7.18, 0.38]	*p* = 0.08	18%
Convalescence								
Sequelae stage	2	3.49 [−2.03, 9.01]	*p* = 0.21	0%	2	−3.78 [−7.61, 0.04]	*P* = 0.05	0%
With or without conventional drugs								
Yes	5	2.02 [−4.70, 8.74]	*p* = 0.56	0%	2	−0.51 [−2.65, 1.63]	*p* = 0.64	0%
No	3	3.71 [−0.06, 7.49]	*P* = 0.05	0%	4	−4.26 [−6.96, −1.55]	*P* < 0.01	0%

#### 3.4.6 Facial disability index-social (FDIS) scores

**Figure 11:**
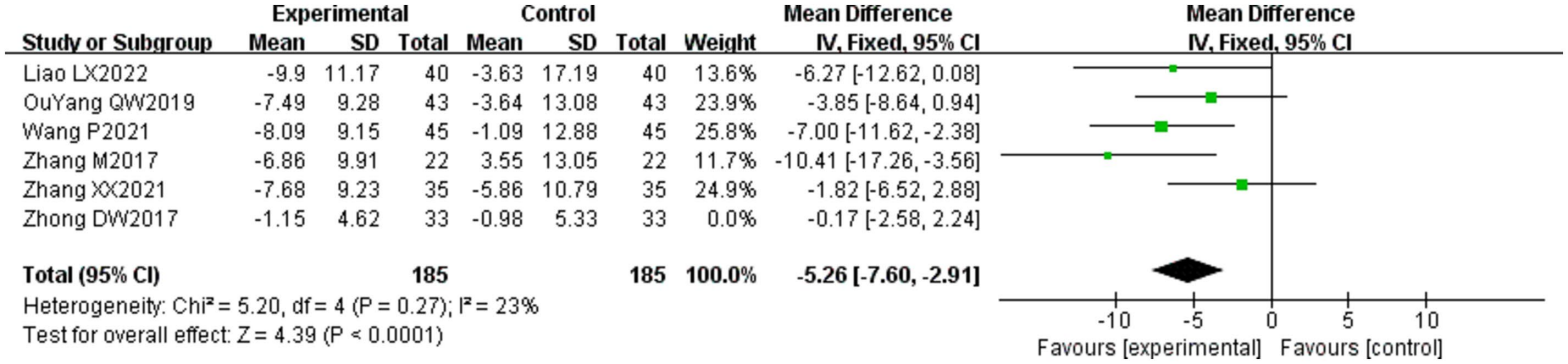
Forest plot of FDIS scores for both groups.

Six of the 36 included papers (20, 37, 43, 50–52) reported changes in FDIP scores before and after treatment of the study participants, and a total of 436 patients were included. Heterogeneity test: (*I*^2^ = 64%, *p* = 0.02), statistically high heterogeneity was observed among the literature, and visual assessment of the funnel plot indicated a slight publication bias (Figure 6E), but the Egger’s test (*p* = 0.47) proved that no statistically significant publication bias was found. Sensitivity analysis showed that this article, Zhong DW2017 (51), made the overall results of FDIS scores unstable (Figure 4E), and after removing this article, heterogeneity was found to be acceptable (*I*^2^ = 23%, *p* = 0.27), and the patients in this study had significantly lower pre-treatment FDIS scores compared to the other five studies, suggesting that the patients included in this study were better functioning in their social lives and had less severe disease, finding a source of heterogeneity. The study was excluded and a fixed-effect model was used (Figure 11). Meta-analysis showed (MD = −5.26, 95% CI [−7.60, −2.91], *p* < 0.001) that the difference in FDIS scores between the experimental group and the control group was statistically significant, indicating that the treatment of the experimental group was better than that of the control group in reducing the FDIS scores of patients with PFP. Subgroup analysis showed that: (1) Grouped by injection method: choosing the method of acupoint injection (MD = −3.81, 95% CI [−6.80, −0.82], *p* = 0.01), the treatment of the experimental group was superior to that of the control group in lowering the FDIS scores of patients with PFP. (2) Grouped by whether or not to cooperate with conventional drugs: without cooperating with the use of conventional drugs (MD = −4.26, 95% CI [−6.96, −1.55], *p* < 0.01), the treatment of the experimental group was superior to that of the control group in lowering the FDIS score of patients with PFP. The remaining subgroups were not statistically significant (Table 3).

#### 3.4.7 Facial nerve conduction velocity

**Figure 12:**
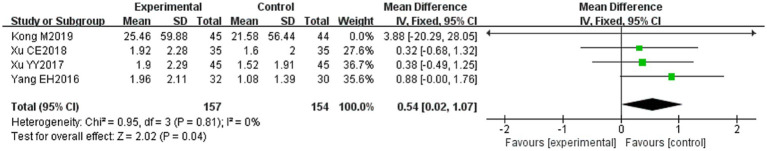
Forest plot of facial nerve conduction velocity in two groups.

Four of the 36 included papers (31–34) reported changes in facial nerve conduction velocity before and after treatment in study participants, with a total of 311 patients included. Heterogeneity test: (*I*^2^ = 0%, *p* = 0.81), there was no statistically significant heterogeneity among the literature, visual assessment of funnel plots indicated a slight publication bias (Figure 6F), and Egger’s test (*p* = 0.19) demonstrated that no statistically significant publication bias was found. The results of the sensitivity analysis were robust (Figure 4F), and a fixed-effects model was chosen (Figure 12). The meta-analysis demonstrated a statistically significant difference in the enhancing of facial nerve conduction velocity between the experimental group and control group (MD = 0.54, 95% CI [0.02, 1.07], *p* = 0.04), indicating superior efficacy of the intervention of the experimental group in enhancing the facial nerve conduction velocity of patients with PFP compared to the control group.

#### 3.4.8 Compound muscle action potential (CMAP) amplitude

**Figure 13:**
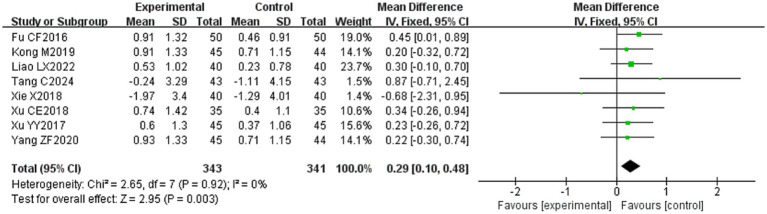
Forest plot of CMAP amplitude for two groups.

Eight (20, 23, 29, 31, 32, 34, 39, 53) of the 36 included papers reported changes in CMAP amplitude before and after treatment of the study subjects, and a total of 684 patients were included. Heterogeneity test: (*I*^2^ = 0%, *p* = 0.92), there was no statistically significant heterogeneity among the literature, visual assessment of the funnel plots indicated no significant publication bias (Figure 6G), and Egger’s test (*p* = 0.24) proved that no statistically significant publication bias was found. The results of the sensitivity analysis were robust (Figure 4G), and a fixed-effects model was chosen (Figure 13). The meta-analysis demonstrated a statistically significant difference in the changes of CMAP amplitude between the experimental and control groups (MD = 0.29, 95% CI [0.10, 0.48], *p* = 0.003), indicating that the intervention of experimental group was superior to the control intervention in improving CMAP amplitude among patients with PFP.

Subgroup analysis showed that (1) Grouped by age: for adults (MD = 1.21, 95% CI [1.17, 1.26], *p* < 0.001), the treatment of the experimental group was superior to that of the control group in improving the CMAP amplitude of patients with PFP. (2) Grouped by injection method: Choosing intramuscular injection (MD = 0.35, 95% CI [0.16, 0.55], *p* < 0.001), the treatment of the experimental group was superior to that of the control group in increasing CMAP amplitude in patients with PFP. (3) Grouped by disease duration: for patients in the acute stage (MD = 0.35, 95% CI [0.16, 0.55], *p* < 0.001), the treatment in the experimental group was superior to the control group in increasing the CMAP amplitude in patients with PFP. (4) Grouped according to whether or not the treatment was combined with conventional drugs: in the case of combining with conventional drugs (MD = 0.36, 95% CI [0.14, 0.58], *p* < 0.01), the treatment of the experimental group was superior to that of the control group in terms of increasing the CMAP amplitude of patients with PFP (Table 4).

**Table 4:** Results of subgroup analyses of CMAP amplitude.

Subgroup	CMAP amplitude			
	Study	MD [95% CI]	*P-*value	*I* ^2^
Population	8	0.29 [0.10, 0.48]	*P* = 0.003	0%
Grouped by age				
Children (≤18 years old)	3	1.17 [1.09, 1.26]	*p* = 0.11	0%
Adults (18 years old)	5	1.21 [1.17, 1.26]	*P* < 0.001	0%
Grouped by injection method				
Intramuscular injection	7	0.35 [0.16, 0.55]	*P* < 0.001	0%
Acupoint injection	1	0.20 [−0.32, 0.72]	*P* = 0.45	
Grouped by disease duration				
Acute stage	7	0.35 [0.16, 0.55]	*P* < 0.001	0%
Convalescence	1	0.20 [−0.32, 0.72]	*P* = 0.45	
Sequelae stage				
With or without conventional drugs				
Yes	5	0.36 [0.14, 0.58]	*P* < 0.01	0%
No	3	0.29 [−0.02, 0.59]	*p* = 0.07	0%

#### 3.4.9 R1 latency

**Figure 14:**
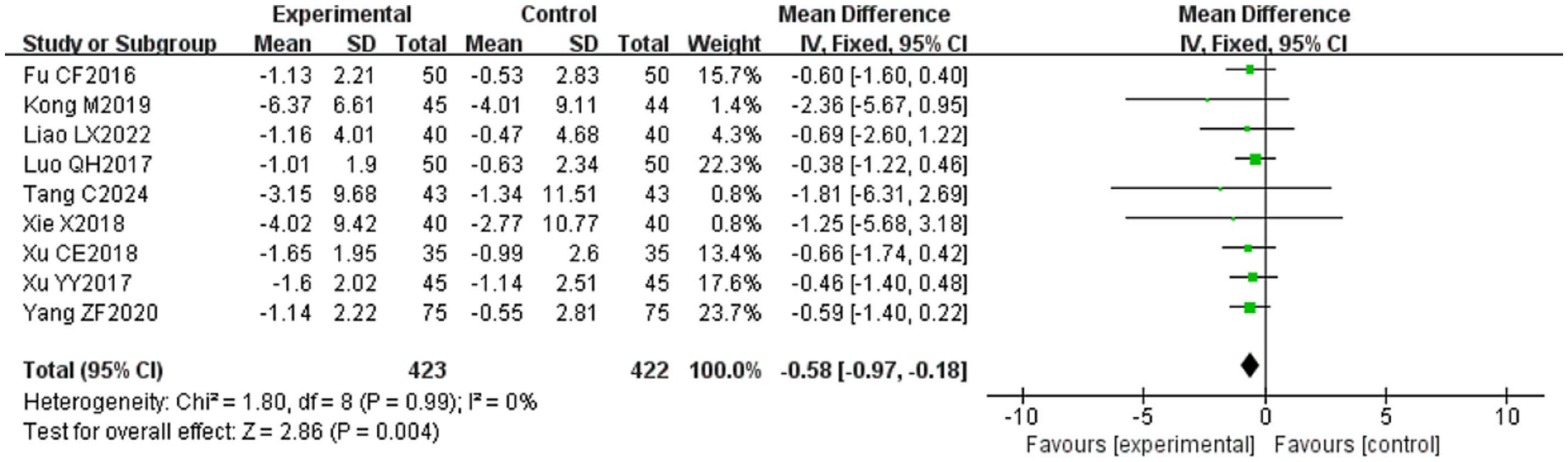
Forest plot of R1 latency for both groups.

Nine (20, 23, 24, 29, 31, 32, 34, 39, 53) of the 36 included papers reported changes in R1 latency before and after treatment of study participants, with a total of 845 patients included. Heterogeneity test: (*I*^2^ = 0%, *p* = 0.99), there was no statistically significant heterogeneity among the literature, visual assessment of the funnel plots indicated a slight publication bias (Figure 6H), and Egger’s test (*p* = 0.72) demonstrated that no statistically significant publication bias was found. The results of the sensitivity analysis were robust (Figure 4H), and a fixed-effects model was chosen (Figure 14). Meta-analysis showed (MD = −0.58, 95% CI [−0.97, −0.18], *p* = 0.004) that the difference in the R1 latency before and after treatment between the experimental group and the control group was statistically significant, suggesting that the treatment of the experimental group was statistically superior to that of the control group in lowering R1 latency in patients with PFP. Subgroup analysis showed that (1) grouped by age: for adults (MD = −0.55, 95% CI [−1.03, −0.07], *p* = 0.02), the treatment of the experimental group was superior to that of the control group in reducing R1 latency in patients with PFP. (2) Grouped by injection method: choosing intramuscular injection (MD = −0.50, 95% CI [−0.89, −0.10], *p* = 0.01), and the treatment of the experimental group was superior to that of the control group in reducing the R1 latency of patients with PFP. (3) Grouped by disease duration: for patients in the acute phase (MD = −0.50, 95% CI [−0.89, −0.10], *p* = 0.01), the treatment of experimental group was superior to the control group in reducing R1 latency in patients with PFP. (4) Grouped according to whether or not it was combined with conventional drug treatment: in combination with conventional drug treatment (MD = −0.48, 95% CI [−0.88, −0.07], *p* = 0.02), the treatment of the experimental group was superior to that in the control group in terms of lowering the R1 latency in patients with PFP (Table 2).

#### 3.4.10 Portmann score

**Figure 15:**
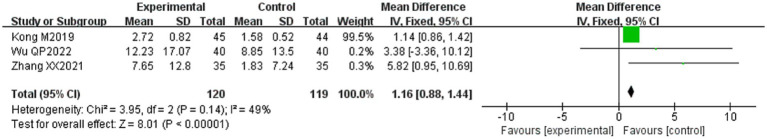
Forest plot of Portmann scores for both groups.

Three (34, 37, 38) of the 36 included papers reported changes in Portmann scores before and after treatment of study participants, with a total of 239 patients included. Heterogeneity test: (*I*^2^ = 49%, *p* = 0.14), the heterogeneity among the literature was statistically acceptable, visual assessment of funnel plots indicated the presence of publication bias (Figure 6I), and Egger’s test (*p* = 0.44) demonstrated that no statistically significant publication bias was found. The results of the sensitivity analysis were robust (Figure 4I), a fixed effects model was chosen (Figure 15). Meta-analysis showed (MD = 1.16, 95% CI [0.88, 1.44], *p* < 0.001) that the difference in Portmann scores was statistically significant when comparing the experimental and control groups before and after the treatment, suggesting that the treatment of the experimental group was statistically superior to that of the control group in improving Portmann score in patients with PFP.

### 3.5 GRADE quality of evidence results

The quality of the evidence was assessed using the GRADE scoring system and assessed using the GRADEpro software. The quality of the evidence was downgraded by one level when there were deficiencies in the design or conduct of each study (e.g., insufficient allocation concealment, lack of blinding); High heterogeneity between outcomes (e.g., *I*^2^ > 50% downgraded by one level and *I*^2^ > 75% downgraded by two grades). The quality of the evidence was downgraded by one level if the population, intervention, control, or outcome (PICO) did not exactly match the clinical question or crossed the threshold for clinical decision on whether to recommend treatment; The quality of the evidence was downgraded by one level if the confidence interval was wide or the sample size was small, resulting in uncertainty in the results; The presence of possible unpublished studies (e.g., funnel plot asymmetry) downgraded the quality of the evidence. The quality of the evidence in this review ranged from moderate to very low, with moderate-quality evidence for the overall effective rate and CMAP amplitude, very low-quality evidence for SFGS dynamic scores, and low-quality evidence for all other outcomes. The main reasons for downgrading were poor allocation concealment or low confidence due to small sample sizes. The specific results are provided in Supplementary Table S4.

## 4 Discussion

The etiology of PFP is still controversial in modern medicine, and viral infection, vascular ischemia, autoimmune inflammatory diseases, and heredity are considered to be the potential causes (55), and the treatment of PFP in modern medicine is mainly through the use of hormonal, antiviral, and nutritive nerve medications (56). The mNGF is the earliest discovered and the most well-studied class of nerve growth factor, which has the biological effects of maintaining and promoting the biological effects of sympathetic nerves and sensory nerve cells from the neural crest in terms of survival, growth and development, and differentiation (57). Acupuncture, as a traditional Chinese medicine therapy, has the effect of improving local circulation, promoting the absorption of edema and inflammatory substances, and reducing facial nerve compression (58), and is often used in the treatment of PFP. In clinical practice, the research of combining acupuncture and mNGF in the treatment of PFP has gradually increased, but there is no relevant Meta-analysis to confirm its clinical efficacy, and whether the combination of the two can enhance the therapeutic effect of PFP is not yet known, therefore, this paper presents a meta-analysis of the clinical efficacy of combining acupuncture and mNGF in the treatment of PFP in order to optimize PFP treatment options.

### 4.1 Major research findings

In this study, the clinical efficacy of acupuncture combined with mNGF in the treatment of PFP was systematically evaluated in terms of eight outcome indicators: overall effective rate, SFGS score, H-B facial nerve function grading score, FDI score, facial nerve conduction velocity, CMAP amplitude, R1 latency, and Portmann score, and a total of 36 RCTs were included, including 2,750 patients. Meta-analysis results showed that acupuncture combined with mNGF in the treatment of PFP had certain advantages in increasing the overall effective rate, FDIP score, facial nerve conduction velocity, CMAP amplitude, Portmann score, and decreasing the SFGS static score, FDIS score, H-B facial nerve function grading score, and R1 latency. Funnel plots and Egger’s test indicated no publication bias. We also conducted sensitivity analyses, which confirmed the stability of the results.

### 4.2 Reasonable interpretation of research findings

In terms of the overall effective rate, acupuncture combined with mNGF treatment for PFP was clinically effective and had better efficacy than either acupuncture or mNGF treatment alone, and subgroup analyses showed that age, the injection method of mNGF, facial paralysis staging, and whether or not to cooperate with conventional medication were not factors influencing the overall effective rate. Therefore, we believe that for patients with PFP of any age and at any stage, the combined application of acupuncture and mNGF can be recommended as a treatment method, and there are no requirements for the injection method or whether it is combined with conventional drugs. Regarding SFGS scores, acupuncture combined with mNGF treatment was better than acupuncture or mNGF treatment alone in reducing SFGS static scores, while the difference in effect was not significant in increasing SFGS dynamic scores. We believe that the possible reasons for this result are that the static scores (such as nasolabial groove symmetry and eyelid fissure width) reflect the recovery of the anatomical structure, while the dynamic scores (such as closing the eyes and puping the cheeks) require the functional reconstruction of the neuro-muscular junction (NMJ), which takes a longer time. All three included studies began treatment in the acute stage of the patients, and the treatment time did not exceed 4 weeks. It is insufficient to complete the reconstruction of the neuromuscular junction function. Therefore, we suggest that the treatment time should be prolonged in order to improve the dynamic score of SFGS. Regarding H-B facial nerve function classification score, acupuncture combined with mNGF treatment was superior to acupuncture alone or mNGF treatment alone in reducing the H-B facial nerve function grading score. This is the same as the result of Sun et al. (59). Regarding FDIP scores, acupuncture combined with mNGF treatment improved FDIP scores better than acupuncture alone or mNGF treatment alone, and subgroup analyses showed that there was no statistically significant difference between the groups. We speculate that the reason for arriving at this result might be that the sample size of a single subgroup is too small, resulting in insufficient statistical power. In the future, personalized and high-quality RCTs studies can be carried out to further verify the research results. In terms of FDIS score, acupuncture combined with mNGF treatment was superior to acupuncture alone or mNGF treatment alone in reducing FDIS score, and subgroup analyses showed that acupuncture combined with mNGF treatment was more effective in reducing FDIS score when acupoint injections were chosen or conventional drugs were not used in conjunction. We believe that since acupoint injection usually selects acupoints on the face, it is conducive to the drug reaching the affected area directly, thereby improving the microcirculation of the patient’s face and repairing the damaged nerve tissue. Therefore, the therapeutic effect is obvious. Good therapeutic effect is positively correlated with the patient’s social willingness. Frequent medication will constantly remind the patient that they are a patient, which thereby causes psychological pressure on patients and reduces their social desires. In the future, clinicians can recommend acupoint injection and drug-free treatment methods to patients with high FDIS scores. In terms of facial nerve conduction velocity, acupuncture combined with mNGF treatment was superior to acupuncture alone or mNGF treatment alone in enhancing facial nerve conduction velocity, which is in line with our expectations. The CMAP represents the summation of action potentials recorded from innervated muscle fibers following peripheral nerve stimulation. CMAP amplitude corresponds to the number of depolarized nerve fibers, reflecting the quantity of functional axons. A reduced CMAP amplitude directly indicates a decrease in the number of functional peripheral nerve axons (60). Regarding CMAP amplitude, the combination therapy of acupuncture and mNGF demonstrated superior efficacy in enhancing CMAP amplitude compared to either acupuncture or mNGF treatment alone. Subgroup analysis revealed that age did not significantly influence the therapeutic effect of the combined approach on CMAP amplitude augmentation. Notably, superior outcomes were observed when the combination therapy was administered intramuscularly, implemented during the acute phase of the condition, or combined with conventional pharmacological interventions. Therefore, we believe that patients with PFP of any age in the acute stage can choose acupuncture combined with intramuscular injection of mNGF and oral conventional drugs in order to increase the amplitude of CMAP. Accurate conduction of electrical impulses along peripheral nerves requires intact function of both axons and myelin sheaths. The R1 latency, a parameter of the blink reflex (BR), serves as a reliable indicator for proximal demyelination and axonal pathology when prolonged (61). Its significant prolongation or absence strongly suggests severe demyelination or axonal disruption (neuropraxia or axonotmesis). Consequently, patients exhibit prolonged recovery times, thereby increasing the risk of persistent sequelae. In terms of R1 latency, subgroup analyses showed that acupuncture combined with mNGF treatment was more effective in reducing R1 latency in adults, patients who chose to use intramuscular injection, patients in the acute phase, and patients who were treated with conventional medications. Therefore, we recommend that adult patients with PFP in the acute stage can choose acupuncture combined with intramuscular injection of mNGF and oral conventional drugs in order to reduce the R1 latency. In terms of Portmann score, acupuncture combined with mNGF treatment was superior to acupuncture alone or mNGF treatment alone in improving the Portmann score. Overall, the combination of acupuncture and mNGF treatment for PFP was clinically effective and more effective than acupuncture alone or mNGF alone. Mechanistically, acupuncture and mNGF may act synergistically to enhance neuroregeneration. We speculate that acupuncture may improve local microcirculation and reduce edema and ischemia at the facial nerve compression site, thereby creating an optimized microenvironment for mNGF to inhibit neuronal apoptosis and stimulate axonal growth, thereby enhancing the delivery of mNGF to damaged nerves. In addition, acupuncture may also optimize the bioavailability of mNGF by upregulating endogenous NGF expression (62), prolong the action time of nerve growth factors, and avoid the limitations of rapid concentration drop after mNGF alone.

## 5 Strengths and limitations

This study is the first systematic evaluation of acupuncture combined with mNGF in the treatment of PFP, which is innovative. The study was conducted in strict accordance with the Cochrane Handbook for Systematic Reviews of Interventions (63) and sensitivity analyses and Egger tests were performed on the results of the study, which showed that the results of the indexes of the study were relatively robust and reliable, and provided evidence-based medical evidence for relevant clinical practice. However, there are limitations in this study: the included studies were all from China, with a limited population, and they were all small-sample studies, lacking large-sample, multicentre clinical studies, which affected the external authenticity of this study; only 21 papers described the randomization scheme in detail, of which seven randomization methods were high-risk; only one paper mentioned blinding, and four were high-risk, while the rest did not mention the implementation of blinding, which had a certain risk of bias; None of the included literature had descriptions of inversion, linkage, and the incidence of facial muscle spasm, nor did they mention follow-up, which made it impossible to evaluate the long-term efficacy of acupuncture combined with mNGF in the treatment of PFP; the selection of acupoints for treatment protocols and the acupuncture technique were not uniform in the various literature, which might be of some heterogeneity; the optimal acupuncture point selection for the treatment of PFP has not been determined; only four literatures reported adverse reactions, making it impossible for us to evaluate its safety. With the exception of the overall effective rate and CMAP amplitude, the quality of the evidence was low or very low.

## 6 Conclusion

The results of our current systematic review and meta-analysis indicated that acupuncture combined with mNGF in the treatment of PFP had certain advantages in increasing the overall effective rate, FDIP score, facial nerve conduction velocity, CMAP amplitude, Portmann score, and decreasing the SFGS static score, FDIS score, H-B facial nerve function grading score, and R1 latency. This article provides new evidence for the treatment of PFP with acupuncture combined with mNGF, which may provide new options for the treatment pathway of PFP in clinical guidelines and optimize individualized treatment options. It can be used as one of the TCM therapeutic strategies for the treatment of PFP. However, due to the limitations of the study, multicentre, large-sample, high-quality RCTs are still needed for its comprehensive evaluation.

## Data Availability

The original contributions presented in the study are included in the article/Supplementary material, further inquiries can be directed to the corresponding authors.
