# Supplementary material for: Acupuncture combined with mouse nerve growth factor in the treatment of peripheral facial palsies: systematic review and meta-analysis
Source: Front Med (Lausanne). 2025 Aug 29;12:1657641. doi: 10.3389/fmed.2025.1657641 (PMC12425716; doi:10.3389/fmed.2025.1657641)
Supplement: Supplementary file 2 [file Table_2.DOCX]

**S2 Table.** Search strategy

1、PubMed search strategy

| Number | Search details |
| --- | --- |
| #1 | ((((((((((((((((((((((((((peripheral facial palsies[MeSH Terms]) OR (Paralyses, Facial)) OR (Paralysis, Facial)) OR (Facial Palsy)) OR (Facial Palsies)) OR (Palsies, Facial)) OR (Palsy, Facial)) OR (Hemifacial Paralysis)) OR (Paralyses, Hemifacial)) OR (Facial Palsy, Lower Motor Neuron)) OR (Facial Palsy, Lower Motor Neuron)) OR (Facial Paralysis, Peripheral)) OR (Facial Paralyses, Peripheral)) OR (Paralysis, Peripheral Facial)) OR (Peripheral Facial Paralysis)) OR (Lower Motor Neuron Facial Palsy)) OR (Facial Palsy, Upper Motor Neuron)) OR (Facial Paralysis, Central)) OR (Central Facial Paralyses)) OR (Central Facial Paralysis)) OR (Facial Paralyses, Central)) OR (Paralyses, Central Facial)) OR (Paralysis, Central Facial)) OR (Upper Motor Neuron Facial Palsy)) OR (Facial Paresis)) OR (Pareses, Facial)) OR (Paresis, Facial) |
| #2 | (((((Acupuncture[MeSH Terms]) OR (Electroacupuncture)) OR (Pharmacopuncture)) OR (acupuncture treatment)) OR (Needling)) OR (needling therapy) |
| #3 | (mouse nerve growth factor) OR (nerve growth factor) |
| #4 | ((((Random) OR (RCT)) OR (controlled clinical trial)) OR (randomized controlled trial)) OR (Randomized) |
| #5 | #1 AND #2 AND #3 AND #4 AND #5 |

2、Embase search strategy

| Number | Search details |
| --- | --- |
| #1 | ('peripheral facial palsy'/exp OR 'facial paralysis'/exp OR 'hemifacial paralysis'/exp OR 'facial paresis'/exp OR 'lower motor neuron facial palsy' OR 'upper motor neuron facial palsy' OR 'central facial paralysis' OR ((peripheral NEAR/3 (facial NEXT/1 palsy* OR facial NEXT/1 paralysis)):ti,ab,kw) OR ((hemifacial OR "lower motor neuron" OR "upper motor neuron") NEAR/3 (paralysis OR palsy)):ti,ab,kw) |
| #2 | ('acupuncture'/exp OR 'electroacupuncture'/exp OR 'pharmacopuncture'/exp OR 'acupuncture treatment':ti,ab,kw OR 'needling therapy':ti,ab,kw) |
| #3 | ('mouse nerve growth factor'/exp OR 'nerve growth factor'/exp OR 'nerve growth factor':ti,ab,kw) |
| #4 | ('randomized controlled trial'/exp OR 'random*':ti,ab,kw OR 'RCT':ti,ab,kw OR 'controlled clinical trial':ti,ab,kw) |
| #5 | #1 AND #2 AND #3 AND #4 |

3、Web of science search strategy

| Number | Search details |
| --- | --- |
| #1 | TS=((("peripheral facial palsy" OR "facial paralysis" OR "facial palsy" OR "hemifacial paralysis" OR "facial paresis") OR ("lower motor neuron facial palsy" OR "upper motor neuron facial palsy" OR "central facial paralysis")) OR (peripheral NEAR/3 (facial NEAR/1 palsy* OR facial NEAR/1 paralysis)) OR (hemifacial NEAR/3 (paralysis OR palsy))) |
| #2 | TS=(("acupuncture" OR "electroacupuncture" OR "pharmacopuncture" OR "acupuncture treatment" OR "needling therapy")) |
| #3 | TS=("mouse nerve growth factor" OR "nerve growth factor") |
| #4 | TS=((random* OR RCT OR "randomized controlled trial" OR "controlled clinical trial")) |
| #5 | #1 AND #2 AND #3 AND #4 |
