# Supplementary material for: Acupuncture combined with mouse nerve growth factor in the treatment of peripheral facial palsies: systematic review and meta-analysis
Source: Front Med (Lausanne). 2025 Aug 29;12:1657641. doi: 10.3389/fmed.2025.1657641 (PMC12425716; doi:10.3389/fmed.2025.1657641)
Supplement: Supplementary file 3 [file Table_3.DOCX]

S3 Table Basic information on included studies

| Basic information on the inclusion of literature | | | | | | | | | | |
| --- | --- | --- | --- | --- | --- | --- | --- | --- | --- | --- |
|  | Number of cases/case | Age (year) | | course of disease（ｘ±ｓ） | | installments | Intervention measure | | Course of treatment/ d | Outcome index |
| literatures | Experimental group/Control group | Experimental group | Control group | Experimental group | Control group |  | Experimental group | Control group |  |  |
| Liao LX 2022[20] | 40/40 | 39.4±5.5 | 38.2±5.3 | 8.7±1.8d | 8.2±1.6d | acute stage | Ⅰ+Ⅳ | Ⅳ | 56d | ①④⑥⑦ |
| Liu CL 2017[21] | 40/40 | 10.23±0.82 | 10.37±0.58 | 5.45±1.23d | 5.27±1.58d | acute stage | Ⅱ+Ⅴ | Ⅱ | 21d | ①② |
| Sheng YX 2015[22] | 38/35 | 42.8±13.5 | 41.6±16.1 | 103.9±36.6d | 104.5±30.3d | convalescence | Ⅱ+Ⅳ+Ⅵ | Ⅱ+Ⅵ | 56d | ① |
| Yang C 2024[23] | 43/43 | 42.11±10.53 | 42.07±10.61 | 3.08±0.95d | 3.43±0.97d | acute stage | Ⅲ+Ⅳ | Ⅳ | 30d | ①⑥⑦ |
| Luo QH 2017[24] | 50/50 | 64.0±5.8 | 67.0±6.4 | 2.6±1.4d | 2.1±1.3d | acute stage | Ⅰ+Ⅳ+Ⅵ | Ⅰ+Ⅵ | 28d | ①⑦ |
| Yuan HQ 2018[25] | 62/58 | 40.6 ± 5.1 | 41.3 ± 5.1 | 11.7 ± 2.3d | 11.5 ± 2.6d | acute stage | Ⅰ+Ⅳ+Ⅵ | Ⅰ+Ⅵ | 28d | ① |
| Shi LG 2016[26] | 43/43 | 37.4±4.7 | 37.4±4.7 | <7d | <7d | acute stage | Ⅰ+Ⅳ+Ⅵ | Ⅰ+Ⅵ | 15d | ① |
| Xue HB 2021[27] | 30/30 | 50.1±16.4 | 51.9±15. | 6.5±3.3  months | 6.9±3.8  months | sequelae stage | Ⅰ+Ⅴ+plum blossom needle | Ⅰ+plum blossom needle | 42d | ① |
| Zou T 2014[28] | 30/30 | 36.32±7.84 | 38.26±6.42 | 2.43±1.32d | 2.33±1.36d | acute stage | Ⅰ+Ⅳ+Ⅵ | Ⅰ+Ⅵ | 28d | ① |
| Yang ZF 2020[29] | 75/75 | 45.26±18.48 | 45.12±18.34 | 4.08±1.85d | 4.13±1.89d | acute stage | Ⅰ+Ⅳ+Ⅵ | Ⅰ+Ⅵ | 28d | ①⑥⑦ |
| Yang SJ 2017[30] | 30/30 | 35.69±14.93 | 37.62±16.37 | 9.37±1.86d | 8.65±1.12d | acute stage | Ⅰ+Ⅴ | Ⅴ | 21d | ① |
| Xu YY 2017[31] | 45/45 | 6.2±1.3 | 6.0±1.5 | 3.2±0.6d | 3.3±0.4d | acute stage | Ⅰ+Ⅳ+Ⅵ | Ⅰ+Ⅵ | 28d | ③⑤⑥⑦ |
| Xu CE 2018[32] | 35/35 | 5.1±1.7 | 5.6±1.3 | 2.3±2.4d | 2.7±2.5d | acute stage | Ⅰ+Ⅳ+Ⅵ | Ⅰ+Ⅵ | 28d | ①⑤⑥⑦ |
| Yang EH 2016[33] | 32/30 | 3.41±2.35 | | 1～7d | 1～7d | acute stage | Ⅱ+Ⅴ+Ⅵ | Ⅱ+Ⅵ | 25d | ①⑤ |
| Kong M 2019[34] | 45/44 | 8.08±1.95 | 5.42±1.17 | 1.21±0.31 months | 1.20±0.33months | convalescence | Ⅰ+Ⅴ | Ⅰ | 21d | ①③⑤⑥⑦⑧ |
| Zhang H 2017[35] | 30/30 | 19-73 | 21-68 | 3-12 months | 4-13months | convalescence | Ⅰ+Ⅴ | Ⅰ | 36d | ① |
| Mo JN 2011[36] | 30/30 | 45.3±3.4 | 43±4.2 | <7d | <7d | acute stage | Ⅰ+Ⅳ+Ⅵ | Ⅰ+Ⅵ | 7d | ① |
| Zhang XX 2021[37] | 35/35 | 41.5±6.9 | 40.9±5.4 | 6～70h | 5～72h | acute stage | Ⅰ+Ⅳ+Ⅵ | Ⅰ+Ⅵ | 7d、14d | ①④⑧ |
| Wu QP 2022[38] | 40/40 | 48.5±1.1 | 48.1±0.9 | 2.6±0.4ｄ | 2.5±0.1ｄ | acute stage | Ⅰ+Ⅳ+Ⅵ | Ⅰ+Ⅵ | 21d | ①⑧ |
| Fu CF 2016[39] | 50/50 | 46.80±18.69 | 44.50±15.20 | 4.15±1.84d | 3.72±1.61d | acute stage | Ⅰ+Ⅳ+Ⅵ+Physical Factors Ultrashort Wave Therapy | Ⅰ+Ⅵ+Physical Factors Ultrashort Wave Therapy | 28d | ①⑥⑦ |
| Li XM 2015[40] | 40/40 | 36.71±3.75 | 33.05±3.64 | 7~14 d | 5~13 d | acute stage | Ⅰ+Ⅳ+Ⅵ | Ⅰ+Ⅵ | 28d | ① |
| Lei CY 2017[41] | 40/40 | average 36 | average 35 | 7-14 d | 5—13d | acute stage | Ⅰ+Ⅳ | Ⅰ | 28d | ① |
| Huang XB2022[42] | 31/31 | 37.52±4.31 | 37.58±4.35 | 3.78±1.45d | 3.54±1.42d | acute stage | Ⅰ+Ⅳ+superlaser | Ⅰ+superlaser | 24d | ①② |
| Wang P 2021[43] | 45/45 | 48.02±3.16 | 47.21±2.89 | 6.93±1.53  months | 6.59±1.19months | Sequelae stage | Ⅰ+Ⅴ | Ⅴ | 28d | ①③④ |
| Zeng H 2020[44] | 45/45 | 48.02±3.16 | 47.21±12.89 | 6.93±1.53  months | 6.59±1.19months | Sequelae stage | Ⅰ+Ⅴ | Ⅴ | 28d | ①② |
| Cai RJ 2018[45] | 38/38 | 9.15±0.68 | 10.23±0.65 | 5.84±0.77d | 6.18±0.63d | acute stage | Ⅰ+Ⅴ | Ⅰ | 21d | ① |
| Wu DH2016[16] | 30/30 | 9-73 | | 3h-3months | 3h-3months | convalescence | Ⅰ+Ⅳ | Ⅰ | 28d | ① |
| Qu D 2018[46] | 18/18 | 5.9±2.65 | 6.38±1.34 | 2.10±3.24d | 3.15±1.5d | acute stage | Ⅰ+Ⅴ+Ⅵ | Ⅰ+Ⅵ | 28d | ① |
| Liu L 2019[47] | 49/49 | 37.8±4.4 | 37.4±4.0 | 7.6±1.9d | 7.2±1.6d | acute stage | Ⅰ+Ⅳ+Ⅵ | Ⅰ+Ⅵ | 28d | ① |
| Wang WJ 2018[48] | 30/30 | 28.82±1.50 | 28.40±1.30 | 48.40±12.20d | 45.40±10.20d | convalescence | Ⅰ+Ⅴ | Ⅰ | 24d | ① |
| Sun LL 2014[49] | 40/40 | 39.33±10.51 | 40.15±9.84 | 2ｄ-6months（average2.6months） | 3ｄ-6months（average2.1months） | convalescence | Ⅰ+Ⅴ | Ⅰ | 14d | ① |
| Ouyang QW2019[50] | 43/43 | 46.2±2.4 | 45.8±2.5 | >3months | >3months | convalescence | Ⅰ+Ⅴ | Ⅴ | 28d | ①③④ |
| Zhong DW 2017[51] | 33/33 | 39.95±4.25 | 40.99±4.02 | —— | —— | —— | Ⅰ+Ⅳ+Ⅵ | Ⅰ+Ⅵ |  | ①④ |
| Zhang M 2017[52] | 22/22 | 44.68±10.78 | 44.00±10.40 | 6.86±3.99months | 8.27±5.16months | Sequelae stage | Ⅰ+Ⅴ | Ⅰ | 28d | ①④ |
| Xie X 2018[53] | 40/40 | average 38.4±6.3 | | 3.6±0.9d | | acute stage | Ⅱ+Ⅳ+Ⅵ | Ⅰ+Ⅵ | 14d | ①⑥⑦ |
| Zhang SH 2018[54] | 26/24 | 17-71 | 15-65 | — | — | — | Ⅰ+Ⅳ+Moxibustion on ginger | Ⅰ+Moxibustion on ginger | 20d | ① |

Note: (1) Conventional drugs include: a. Anti-inflammatory drugs: e.g. dexamethasone, etc.; b. Nutritional nerve drugs: e.g. vitamin B1, etc.; (2) Ⅰ：acupuncture；Ⅱ：electroacupuncture; Ⅲ：Warm Needling； Ⅳ：Mouse nerve growth factor intramuscular injection； Ⅴ：Mouse nerve growth factor acupoint injection；Ⅵ：conventional drugs. (3) ① Overall effective rate ② SFGS score ③ H-B score ④ FDI score ⑤ Facial nerve conduction velocity ⑥ CMAP amplitude ⑦ R1 latency ⑧ Portmann score
