# Supplementary figures and images for: Acupuncture combined with mouse nerve growth factor in the treatment of peripheral facial palsies: systematic review and meta-analysis
Source: Front Med (Lausanne). 2025 Aug 29;12:1657641. doi: 10.3389/fmed.2025.1657641 (PMC12425716; doi:10.3389/fmed.2025.1657641)

**S4 Table** GRADE quality of evidence results


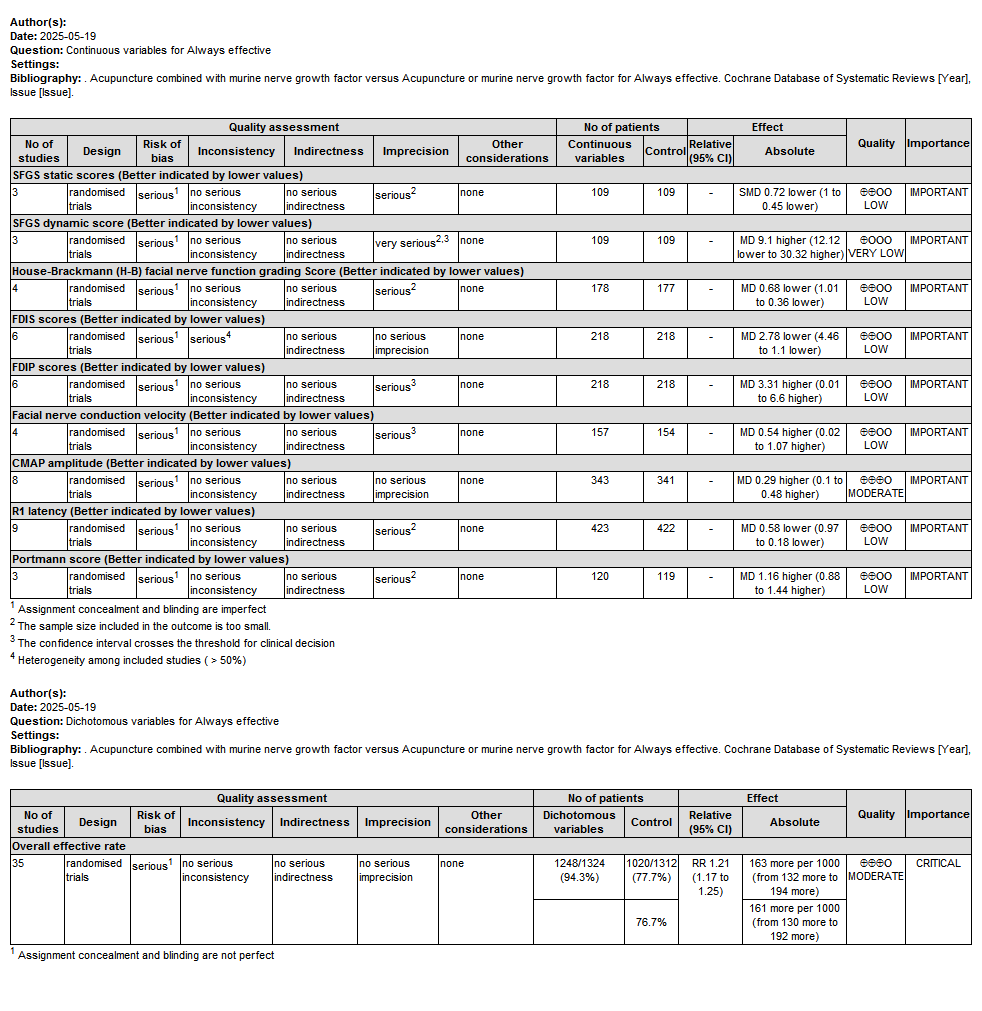

Supplement: Supplementary file 4 [file Table_4.DOCX]
