# Supplementary material for: Acupuncture combined with mouse nerve growth factor in the treatment of peripheral facial palsies: systematic review and meta-analysis
Source: Front Med (Lausanne). 2025 Aug 29;12:1657641. doi: 10.3389/fmed.2025.1657641 (PMC12425716; doi:10.3389/fmed.2025.1657641)
Supplement: Supplementary file 5 [file Table_5.DOCX]

| Full name | Definitions |
| --- | --- |
| Acupuncture | Acupuncture, also known as needling or acupuncture therapy, is a traditional Chinese medical treatment method that involves stimulating specific points on the body with needles to regulate qi and blood, balance yin and yang, and promote self-healing of the body(Including but not limited to acupuncture, triangular acupuncture, skin acupuncture, plum blossom acupuncture, intradermal acupuncture, fire acupuncture, awn acupuncture, electroacupuncture, warm needling, thread embedding therapy and other related acupuncture therapies). |
| Mouse nerve growth factor（mNGF） | The full name is mouse nerve growth factor for injection, which is a bioactive protein extracted from the submandibular gland of mice, and is a bioactive protein with a relative molecular weight of 2.65×10^4^. It is one of the most important bioactive protein substances in the nervous system, and is an essential protein molecule for the differentiation, development and maintenance of normal function of the central and peripheral nervous system, which can promote the repair of the nervous system after injury. |
| Acupoint injection | Acupoint injection is a treatment method in which a medicinal solution is injected into acupuncture points to prevent and treat diseases. It can combine acupuncture stimulation with the performance of drugs and the penetration of acupuncture points to exert its comprehensive effect, so it has a special effect on some diseases. |
| Intramuscular injection | Intramuscular injection is a method of injecting a dose of liquid medication into muscle tissue. |

**S5 Table** Definitions of treatments that appear in the text
